# Average Weight of Children at Birth

**Published:** 1874-03

**Authors:** John H. Booth

**Affiliations:** Tally-Hoe, N. C.


### AVERAGE WEIGHT OF CHILDREN AT BIRTH.

BY JOHN H. BOOTH, M.D., OF TALLY-HOE, N. C.

Having long been impressed with the opinion from actual observation of several hundred new born children, a good many of which I had weighed, that children in this country are much heavier than the authorities give, I determined some time since to set down the .weights of such as I could, impartially, and as they came.

I have not been able to weigh all that I have delivered during the time of observation, on account of the difficulty of getting facilities for weighing at some places, while the superstition of the their friends have prevented it at others.

To show the difference between my observation and that of the best authors, I will give a few of the latter.

The reason of the difference, I think, will be obvious if we recollect that these children were neither very few if any exceptions, born in hospitals and in large cities, where the hygienic surroundings of the mother were not favorable to large fœtal developments.

Sir J. Y. Simpson says that the average weight of fifty boys born at the Maternity Hospital at Edinburgh, was seven pounds nine ounces, one drachm; the average weight of fifty girls was six pounds and twelve ounces.

Dr. Joseph Price found the average weight of boys at birth to be seven pounds, five ounces and seven drachms, and that of girls six pounds, eleven ounces and six drachms.

In Germauy, Rhoedeser found the weight, in one hundred and thirteen cases, vary from seven to eight pounds, and says that it is rarely less than six pounds.

Dr. Macauley examined the bodies of several thousand new born and perfect children, at the British Lying-in Hospital, the weight of the smallest being about four pounds, the largest eleven pounds and two ounces. The greatest number was from five to eight pounds. "Take then the average weight of both sexes, it will be found that twelve males weigh as much as thirteen females."

Dr. Clark, of Dublin, found the weight to vary from four to eleven pounds.

While we see authentic accounts of children weighing from fourteen to seventeen pounds occasionally, M. Velpeau says that a new born child of eight or nine pounds weight, is enormous, and indirectly calls in question the veracity of those who affirm they have witnessed much greater weights.

Dr. Ramsbotham, Jr., never saw one that weighed over twelve pounds. Dr. Merriman delivered one weighing fourteen pounds; it was still-born, and Dr. Craft weighed one alive that weighed fifteen pounds. M. Cazeaux gives the average at six to seven pounds, and speaks of some recorded weights of eighteen, twenty, twenty-four and thirty pounds as wonderful exagerations. The most voluminous, of three thousand children born under his charge, at Hotel Dieu, or at La Clinque, weighed ten pounds, and he says it was enormous. Of four thousand born at La Maternite, one only weighed twelve pounds. "*Lachapelle*," (Cazeaux), he mentions a surgeon at Haymoon, who saw a still born child that weighed seventeen pounds, and finally M. Cazeaux delivered a dead child by twining in consultation, that weighed eighteen pounds, and was probably a ten months' child.

I have thus given the experience of authors of the most extensive opportunities for observation.

There can scarcely be found a practitioner of standing in this part of our State, who has not delivered a child weighing at least fifteen pounds. A midwife in this neighborhood, delivered a negress of a child the last winter, that weighed sixteen pounds, and a white woman was delivered of two living male children, four years since, weighing twelve pounds each, on the same plantation.

The first white child that I ever delivered, weighed twelve pounds, and a few months after its birth, I delivered a woman of a boy weighing fifteen pounds, and measuring eight inches across the shoulders. It was still born. But a couple of practitioners had delivered the same woman a few years previously of a male child weighing fourteen pounds and some ounces. The breech presented and it was delivered instrumentally.

Of thirty-seven new born infants, twenty-one males and sixteen females, the weights of which I have recorded, the average weights of the males is eight pounds, thirteen ounces and eleven drachms; of the females seven pounds and seven ounces. The largest male weighed fourteen pounds, and the smallest six pounds. None of the others except one weighed less than seven pounds and a half. The largest female weighed nine pounds and a half, and the smallest five pounds and three quarters.

The mothers of these children generally lived in the country, and led active lives.

The mother of the smallest boy was sedentary, and had been kept in bed, on the lightest diet, for several of the last weeks of her pregnancy, on account of some fancied danger of uterine hemorrhage.

The mother of the largest male child had given birth to two living children previous to its birth, and had had several abortions, at each of which she had flooded fearfully. Consequently during most of the time in the interim she was very feeble and anæmic, all the result of a uterine disease, for which her husband refused to have her treated, except a short time once, by a quack.

The child that weighed fifteen pounds, not in my average, was alive after the head passed, but died before the shoulder could be delivered, on account of their long detention. With my present experience I think I could have saved it. No consultation could be had in time, and I had seen only two cases of labor before.

The placenta was retained by hour glass contraction, and while I was insinuating my fingers between it and the uterus, she being a powerful woman, seized me by the beard, which was long and extremely tender. Forgetful of everything but my agonized chin, I suddenly and incontinently withdrew my hand, leaving the placenta, which however followed, and the case terminated favorably. On my visit the next day, I found my patient sitting up, contrary to my advice, gossiping with half a dozen neighbors, and dipping snuff.

This lady had cataract in both eyes afterwards, and three years later died of diabetes melitus, after having given birth to another large male child which was cyanotic.

